# In vivo and in vitro toxicity profile of tetrabutylammonium bromide and alcohol-based deep eutectic solvents

**DOI:** 10.1038/s41598-023-28928-y

**Published:** 2023-01-31

**Authors:** Shamaila Inayat, Sajid Rashid Ahmad, Sana Javaid Awan, Muhammad Nawshad, Qurban Ali

**Affiliations:** 1 College of Earth and Environmental Sciences, Quaid e Azam Campus, University of the Punjab, Lahore, Pakistan; 2 Zoology Department, Kinnaird College for Women, Lahore, Pakistan; 3 Institute of Basic Medical Sciences, Khyber Medical University, Peshawar, KPK Pakistan; 4 Department of Plant Breeding and Genetics, University of the Punjab, Lahore, Pakistan

**Keywords:** Microbiology, Antimicrobials

## Abstract

Deep eutectic solvents (DESs) have emerged as new promising solvents in the field of “green chemistry,” which possess a broad range of potential applications. However, the ecotoxicological profile of these solvents is still poorly known. In this study, ammonium-based deep eutectic solutions with glycerol (2:2), ethylene glycol (1:2), and diethylene glycol (1:2) as hydrogen bond donors in 1:2 proportion were evaluated for their interaction with various biological systems, including gram-positive and negative bacteria, fungi, fish, and human fibroblast cell lines. The DES synthesis was confirmed by Fourier transform infrared spectroscopy analysis, which analyses the interactions between DES precursors for their synthesis. The antimicrobial activity of tetrabutylammonium bromide: ethylene glycol was the most potent, while tetrabutylammonium bromide: diethylene glycol had a higher LC50 against *C*. *carpio* fish. Tetrabutylammonium bromide: glycerol was supposed to be the most suitable DES in terms of cell viability percentage (118%) and 2,2-diphenyl-1-picrylhydrazyl scavenging activity (93%). Finally, tetrabutylammonium bromide in glycerol can be considered an eco-friendly solvent due to its lower toxicity in both in vivo and in vitro environments.

## Introduction

Deep Eutectic solvent is a combination of two or more chemicals that result in a particular chemical composition that freezes at a specific lower temperature than any other mixture. The formation of Eutectic solvent depends upon the administration of the following factors: (a) the chemicals must form a homogenous mixture in a liquid state which must be immiscible in the solid state, (b) Close interaction is necessary to take place between the starting materials of eutectic solvents for inducing the melting temperature (T_m_) depression, (c) the chemicals must have chemical bonds so that they can combine to form physical bonds such as hydrogen bonding, etc., (d) only those molecules can form eutectic solvents which are in accordance to the altered VantHoff’s equation^1–3^. Green solvents such as DESs must meet a set of criteria to be classified as green media, including non-toxic effects, biodegradability, and a lower cost than other solvents. These solvents are usually liquid at temperatures below 100 °C^4,5^. The main advantage of using DESs is their economic value, as they are much safer and cheaper than ionic liquids. The toxicity level of DESs is quite low, and they are usually prepared from naturally occurring and environmentally friendly elements^6,7^. These natural components have attracted a lot of attention since they are low in toxicity and easier to handle^8^.

Although deep eutectic solvents have a low toxicity range, their characterization and applications are still less known and understood. In a study by Abbott et al., a comparison was devised among the starting materials and DESs, revealing that DESs are moderate in toxicity and overcompensated by the acid concentration. They can also give many toxic results compared to ionic liquids, and their common examples are cholinium dihydrogen citrate, cholinium lactate, etc. According to the study, further research work is required to explain the toxicity and biodegradable nature of the DESs, which are a significant part of green chemistry^9^. Hayyan et al. examined the toxicity and cytotoxicity of ChCl- and phosphonium-based DES. They checked cytotoxicity on brine shrimps and toxicity on bacteria (i.e., *Escherichia coli, Bacillus subtilis, Staphylococcus aureus, and Pseudomonas aeruginosa*). The results revealed that the tested DESs had much higher cytotoxicity and antibacterial activity against studied bacteria than their components^10^. Radosevic and coworkers conducted extensive research on the toxicological effects of three ChCl-based DES, including fish, human cell lines, and wheat. They also evaluated the biodegradable nature of DES by applying it to sewage-containing microorganisms. DES posed low cytotoxicity and no phytotoxicity towards seed germination^11^. Zhao et al. used 20 NADES against *S. aureus, Listeria monocytogenes, E. coli,* and *Salmonella enteritidis*; it was found that acid-containing DESs showed toxicity while all the other solvents were harmless against bacteria^12^. Ghaedi et al.^13^ tested certain DES upon *E. coli* and *L. monocytogenes*. Their results revealed that the toxicity of DES increased by increasing alkyl chain length on HBD on *E. coli,* but no effect was seen on *L.* monocytogenes. There was no relationship between the toxicity of DES and molar ratios. Furthermore, it was found that the type of HBD has a dominant effect on the toxicity of DESs compared to the type of salt^13^. Wikene et al.^14^ studied the antimicrobial properties of natural deep eutectic solvents in which the microorganisms including *E. coli* and a fungus were exposed to NADES. It was observed that the toxicity in *E. coli* was due to the pH of the solutions^14^. In another study by Kristina et al.^15^, natural deep eutectic solvents had been formed by choline chloride as a hydrogen bond acceptor and acids, amides and alcohols as hydrogen bond donors. They assessed the toxicity of these NADES against bacteria such as *E. coli, S. aureus, Proteus mirabilis, P. aeruginosa* and *Salmonella typhimurium* and human cell lines including MCF-1, HEK293T and HeLa cell lines and NADES comprised of acids found more toxic than others^15^. A small library of some novel highly substituted pyrido [2,3-d] pyrimidine derivatives was used to perform multicomponent synthesis, which was mediated by deep eutectic solvents. Some of the synthesized derivatives presented remarkable antifungal and antibacterial agents^16^. In another study by Juneidi et al., ten DESs had been synthesized with both acidic and basic hydrogen bond donors. These DESs were tested against fungi (*Lentinus tigrinus, Aspergillus niger, Phanerochaete chrysosporium and Candida albicans*) and fish (*C. carpio*), it was found that the DESs with basic HBDs had more toxicity than acidic ones^17^. Fahim et al. performed the synthesis, antimicrobial evaluation, molecular docking, and theoretical calculations of novel pyrazolo[1,5-*a*]pyrimidine derivatives on *E. coli, Streptococcus pneumoniae, A. flavus,* and *Geotrichum candidum*. Some of the newly synthesized compounds exhibited promising antimicrobial activities^18^.

In the present study, three deep eutectic solvents were synthesized by combining tetrabutylammonium salt and alcohols such as glycerol, ethylene glycol and diethylene glycol and their synthesis was confirmed by FTIR analysis. The toxicological assays of DESs were conducted against four bacterial strains (Gram-negative, i.e., *E. coli a*nd *P. aeruginosa* and Gram-positive, i.e., *S. aureus* and* L. monocytogenes),* two fungal strains (*A. niger* and *C. albicans*), *Cyprinus carpio* fish and mouse embryonic fibroblast cell lines (NIH3T3). Moreover, the free radical scavenging activity of deep eutectic solvent was also assessed by 2,2-diphenyl-1-picrylhydrazyl (DPPH) assay.

## Results and discussion

**Figure 1:**
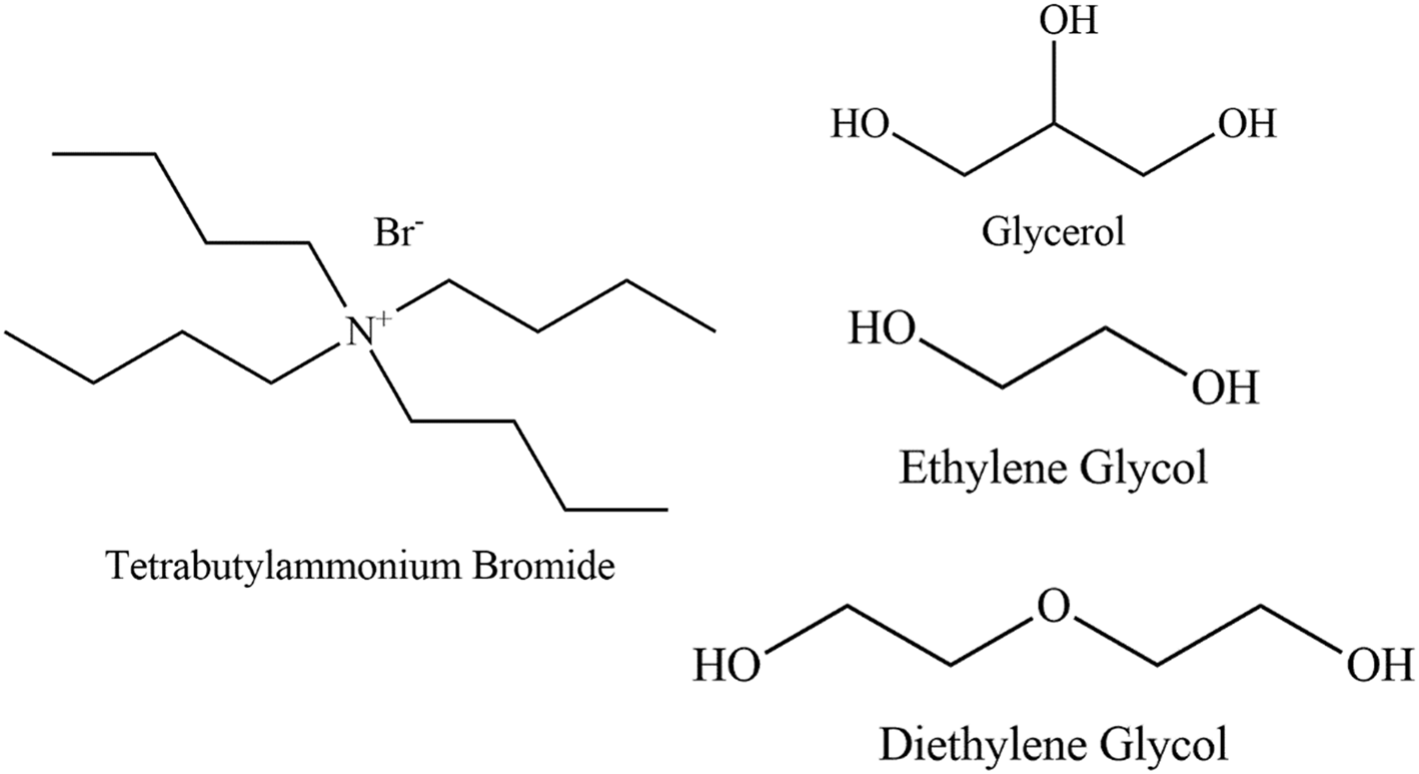
Structure of tetrabutylammonium bromide and alcohols used in this study.

The deep eutectic solvents, tetrabutylammonium bromide: glycerol, tetrabutylammonium bromide: ethylene glycol, and tetrabutylammonium bromide: diethylene glycol were synthesized as homogenous liquids without formation of any precipitates at ambient temperatures. The deep eutectic solvents were formed by the intermolecular interactions of the precursors, such as Van der Waal forces, hydrogen bonding and electrostatic interactions (Fig. 1).

### FTIR analysis

**Figure 2:**
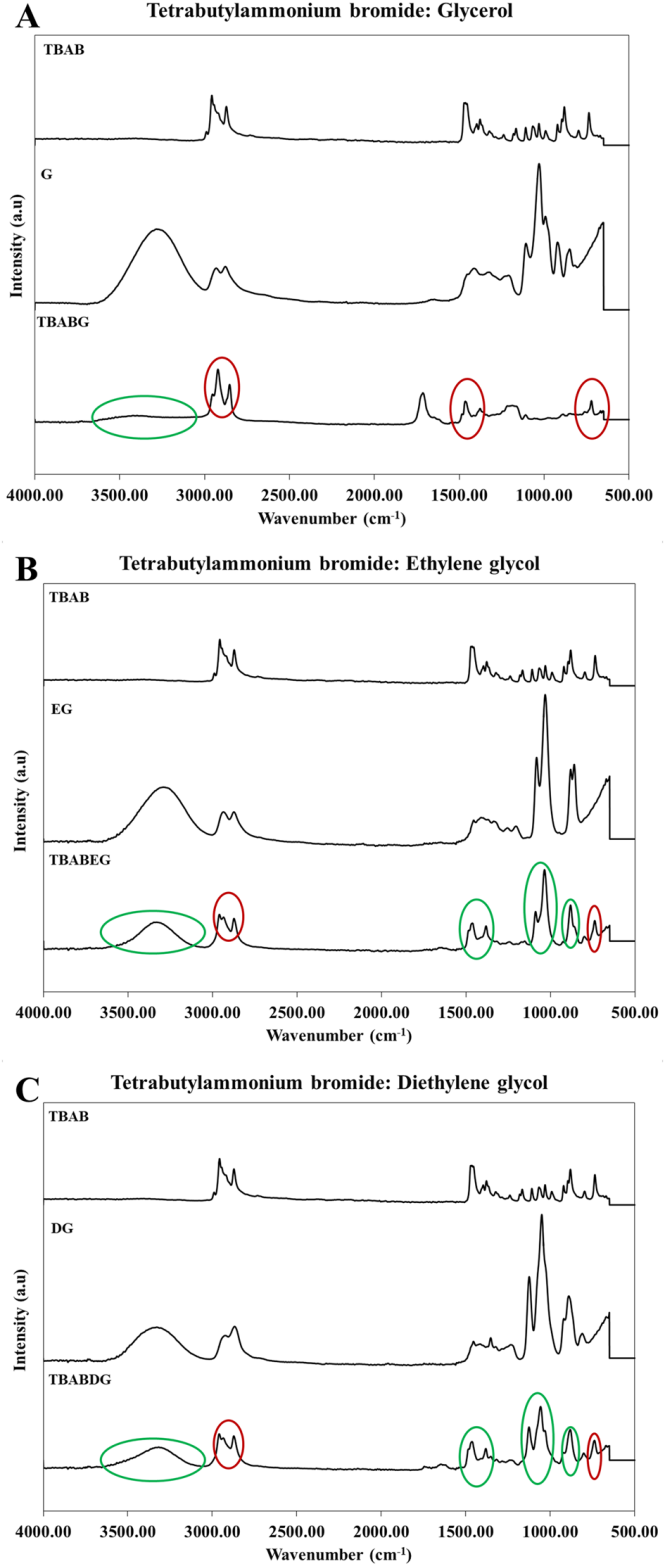
FTIR analysis of DESs and their precursors. (**a**) Synthesized TBABG DES and its precursors, tetrabutylammonium bromide (TBAB), with glycerol (G). (**b**) Synthesized TBABEG DES and its precursors, tetrabutylammonium bromide (TBAB), with ethylene glycol (EG). C) Synthesized TBABDG DES and its precursors, tetrabutylammonium bromide (TBAB), with diethylene glycol (DG). The green circle represents regions similar to hydrogen bond donors, while the red circle represents peaks related to ammonium salts in all DESs.

In synthesising new solvents, it is important to study the existing functional groups, the combination of various substances, or the likely changes in their structure. For this purpose, FTIR was used to analyze the interactions of functional groups in DESs formed along with their reactants. This will also be important in studying the possible structures of the DESs formed for further studies^19^. The individual reactants included Tetrabutylammonium bromide (TBAB), Glycerol (G), Ethylene glycol (EG) and Diethylene glycol (DG). The presence of ammonium salt (TBAB) is indicated by the presence of the N–H bond, giving two peaks at 2957.09 and 2871.86 cm^−1^, that may interlock with other bonds such as (OH and CH)^20,21^ (Fig. 2). The broader peak at 3301.90 cm^−1^ indicated the presence of three hydroxyl groups indicating glycerol, and the smaller peak at 2923.48 cm^−1^ indicated the presence of a C-H bond (Fig. 2a). The peak is slightly smaller because of the presence of one hydrogen compared to ethylene glycol, where two hydrogens are present. In the FTIR spectrum of TBABG, the broader spectrum at 3302.87 cm^−1^ indicated the presence of OH, which is affiliated with glycerol, along with that, the peak at 2922.76 cm^−1^ indicated the presence of the N–H group of TBAB in addition to peaks at 1550 cm^−1^ and 709 cm^−1^ (Fig. 2a). A broad peak is obtained at 3346.73 cm^−1^ which indicated the presence of two OH groups and 2935.57 cm^−1^ referred to the C-H bond of ethylene glycol^22^ (Fig. 2b). A broad peak is obtained at 3346.73 cm^−1^, indicating the presence of OH groups in ethylene glycol. However, the presence of ammonium salt (TBAB) is indicated by the presence of peaks at 2959.45 and 2873.04 cm^−1^ of the N–H bond^23^. Another proof of the presence of salts in the prepared DES is the absorbance at 800–600 cm^−1^ representing the presence of the halide ions (Br and Cl)^21,24^ (Fig. 2). The diethylene glycol IR spectrum presented a peak at 3328.16 cm^−1^ due to OH stretching of intermolecular hydrogen bonding. Moreover, the presence of sharp stretches in the 1300–1000 cm^−1^ region indicated the presence of C–O–C, which was specified as diethylene glycol (Fig. 2c).

### Antimicrobial potential of synthesized DESs

**Figure 3:**
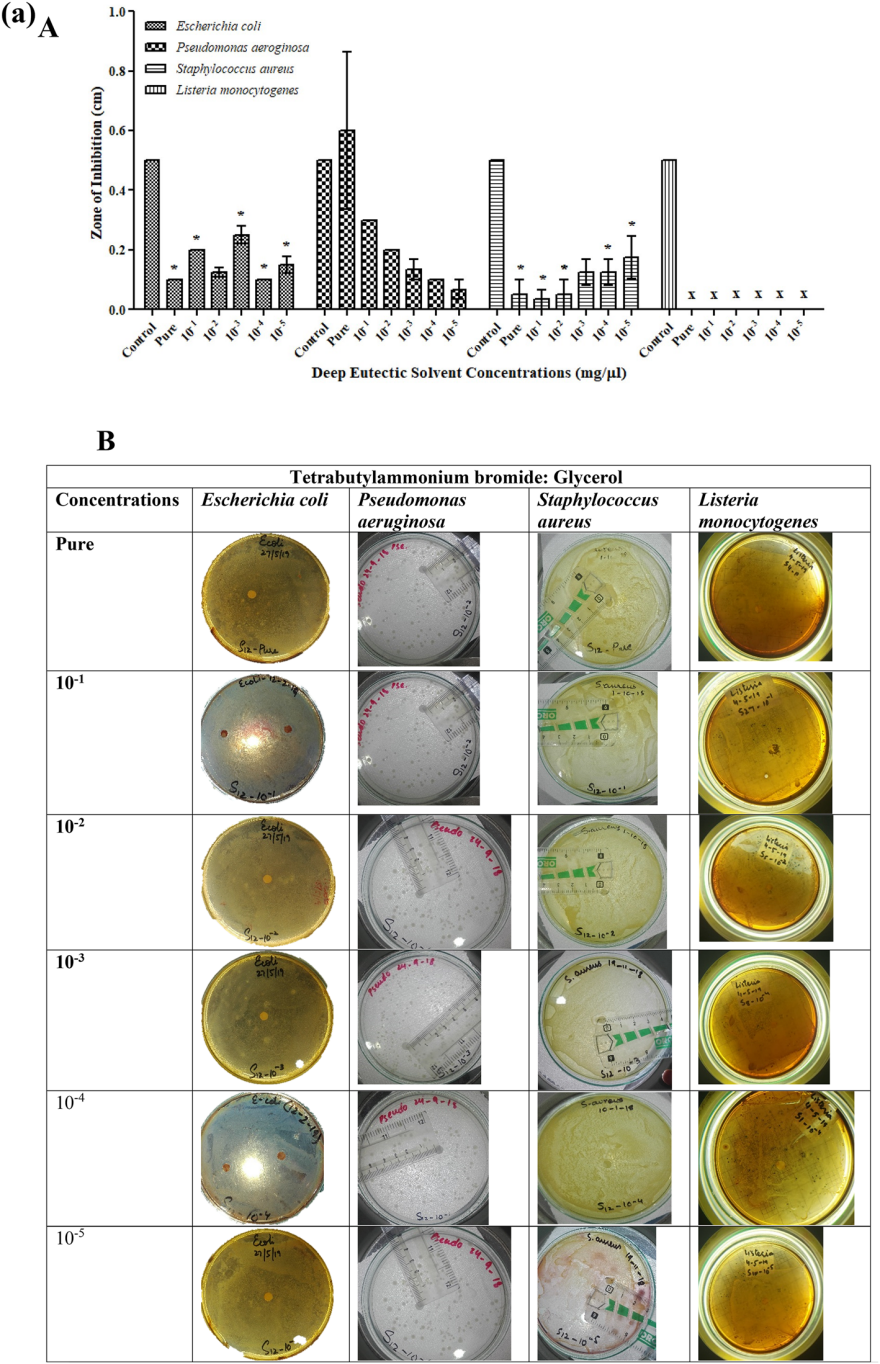

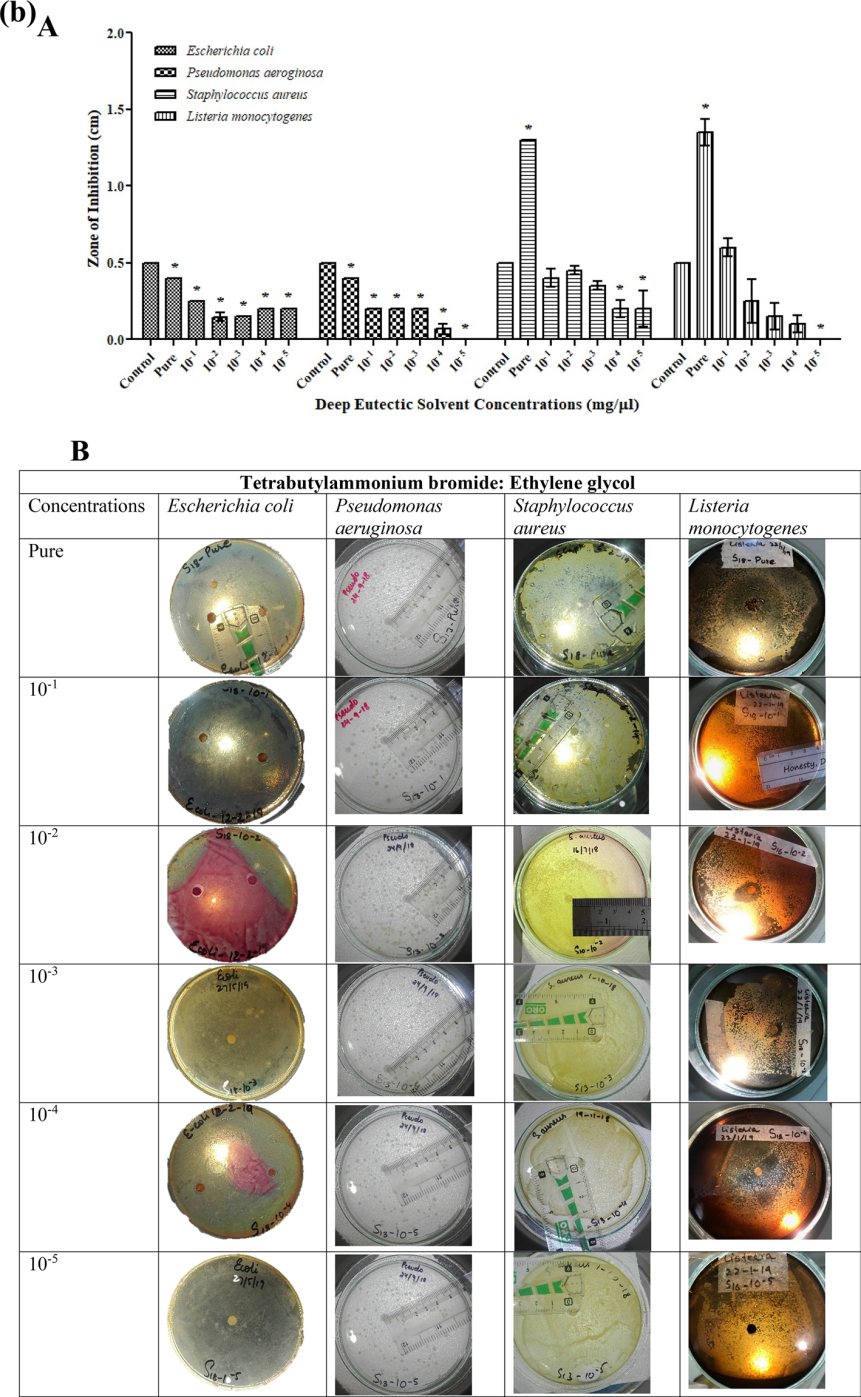

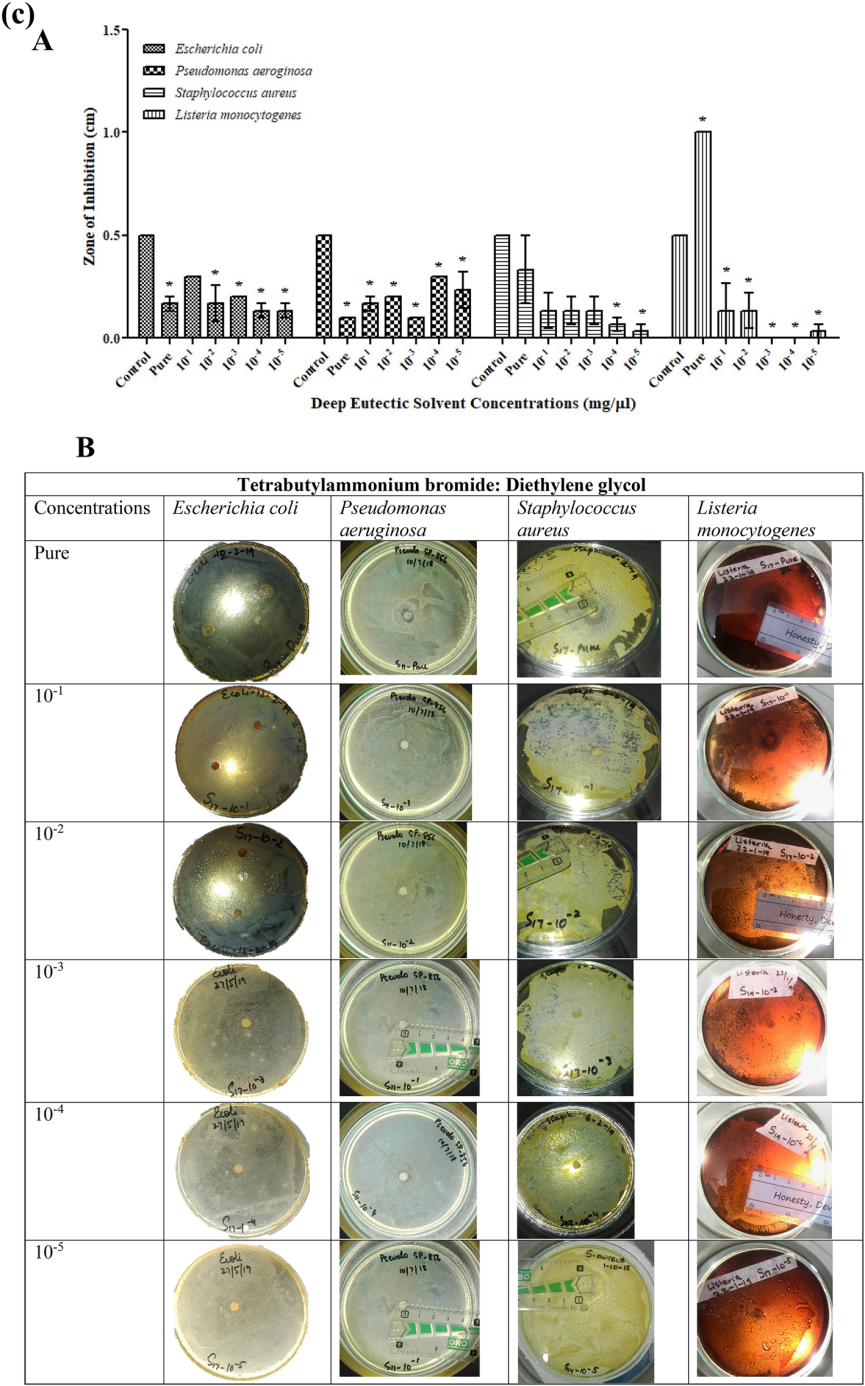
(**a**) Antibacterial activities of TBABG against *E. coli, P. aeruginosa, S. aureus* and *L. monocytogenes*. **A**. TBABG in different concentrations (pure, 10^−1^ to 10^−5^). The *p*-value < 0.05 was considered significant and represented by *, while ^x^ implied no statistical analysis was applied. **B**. Zones of inhibition presented by TBABG against all tested bacterial strains. (**b**) Antibacterial activities of TBABEG against *E. coli, P. aeruginosa, S. aureus* and *L. monocytogenes*. **A**. TBABEG in different concentrations (pure, 10^−1^ to 10^−5^). The *p*-value < 0.05 was considered significant and represented by *, while ^x^ implied no statistical analysis was applied. **B**. Zones of inhibition presented by TBABEG against all tested bacterial strains. (**c**) Antibacterial activities of TBABDG against *E. coli, P. aeruginosa, S. aureus* and *L. monocytogenes*. **A**. TBABDG in different concentrations (pure, 10^−1^ to 10^−5^). The *p*-value < 0.05 was considered significant and represented by *, while ^x^ implied no statistical analysis was applied. **B**. Zones of inhibition presented by TBABDG against all tested bacterial strains.

DESs prepared were excellent due to their homogeneous property. These liquids had a lower melting point than the components used in preparation. All the studied solvents prepared were ammonium-based, paired with Bromide. This study indicates that ammonium-based DESs have toxicological effects on the growth of microorganisms. The antibacterial activity of the investigated DESs against the most frequent pathogenic bacterial and fungal strains, as assessed by a disc diffusion assay, is shown in Table S1. Most of the bacteria studied were suppressed by both DESs, demonstrating that the generating components of DESs did not affect their antimicrobial action. The selected microbial strains were evaluated for the assessment of antimicrobial potential of synthesized DESs (TBABG, TBABEG & TBABDG) (Table S1). According to the observed results, the DES formulations showed antibacterial activity against both gram-negative and gram-positive bacteria. TBABEG pure solvent possesses maximum antibacterial potential against *S. aureus* and *L. monocytogenes* as compared to the other two strains (Fig. 3b). Previously it was assumed by Kristina et al*.* that DES interacts with the peptidoglycan layer of the bacterial cell wall and exhibits toxic effects. Hence Gram-positive bacteria show more toxicity towards DES, and it was assumed that it is due to the lipopolysaccharide membrane covering the cell wall of gram-negative bacterium^15^. This can be supported by a study conducted by Anil et al.^24^, in which toxicity and enhanced drug solubility profile of deep eutectic solvents were assessed. The study revealed that the DESs containing ethylene glycol as hydrogen bond donors had higher toxicity towards tested bacteria than the DESs containing glycerol. The best reason for this could be the somewhat fluidic character of DES medium containing ethylene glycol, which gives an optimum viscosity compared to the high viscosity of glycerol-containing solvents^25^. A study conducted by Wen et al.^25^ found that among four choline chloride-based DESs, which contain urea, acetamide, ethylene glycol and glycerol as hydrogen bond donors, choline chloride (ChCl)-ethylene glycol presented the highest toxicity. The results implied that DESs are non-toxic to bacteria at low doses but have a significantly stronger antibacterial activity at larger concentrations than their components. The bacterium's effective concentration values differed depending on the salt and HBD used in the DES, with ChCl-based DESs having greater EC50 values, particularly when ethylene glycol was used^26^. Likewise, TBABG pure solvent also had higher antibacterial properties against *S. aureus* (Fig. 3a). Moreover, TBABG and TBABDG had more overall toxicity for gram-negative bacteria than gram-positive bacteria in all concentrations used for antibacterial assay (Fig. 3a,c).

**Figure 4:**
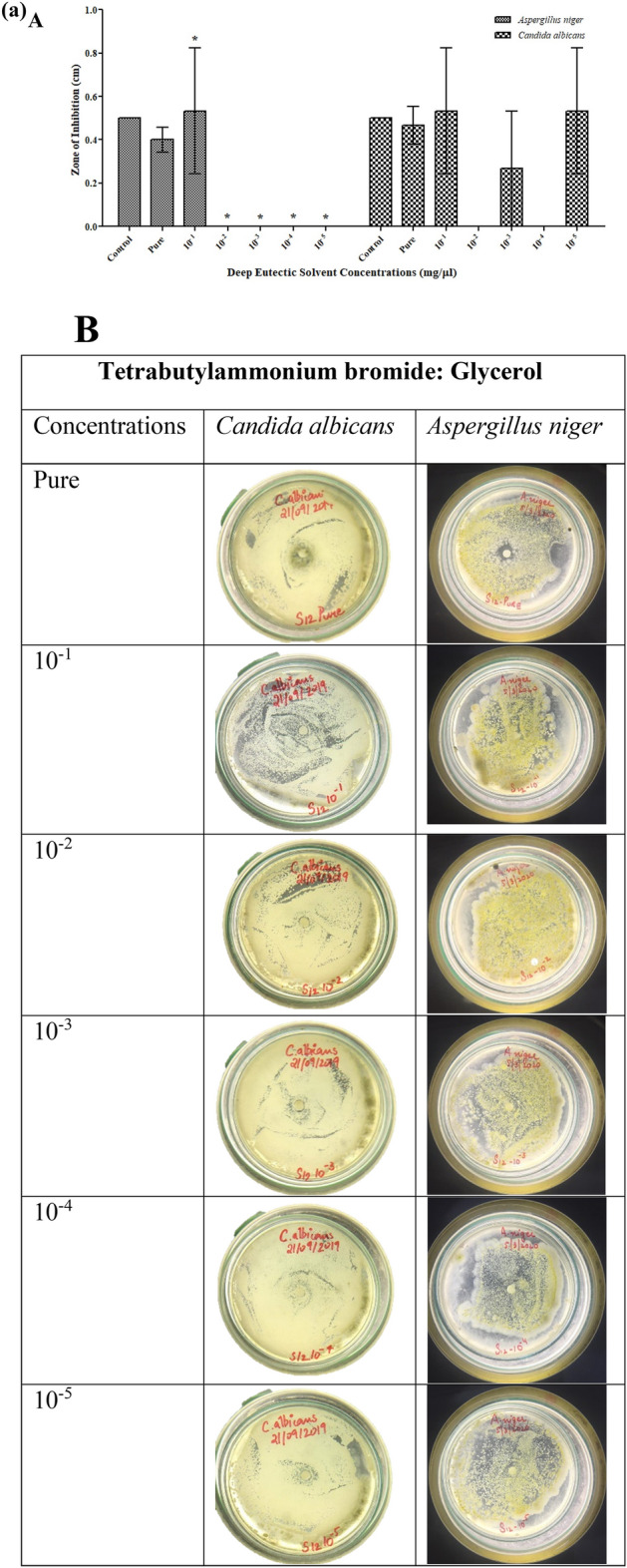

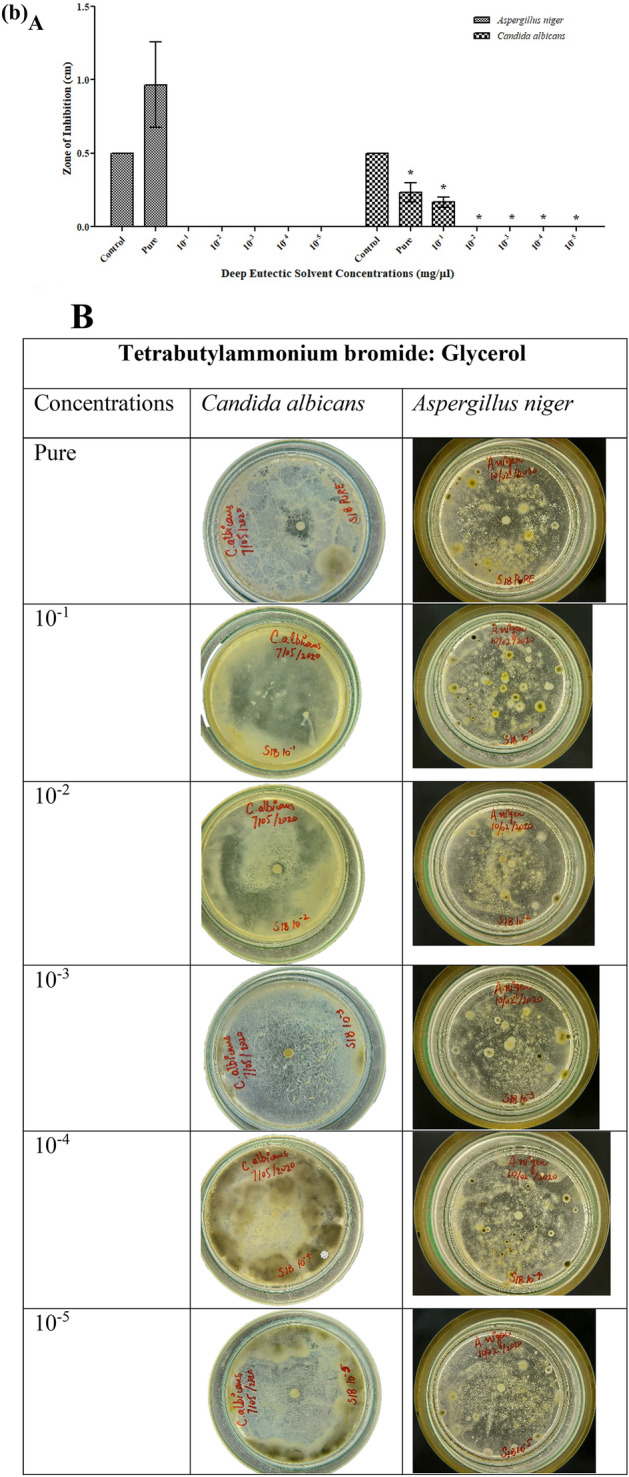

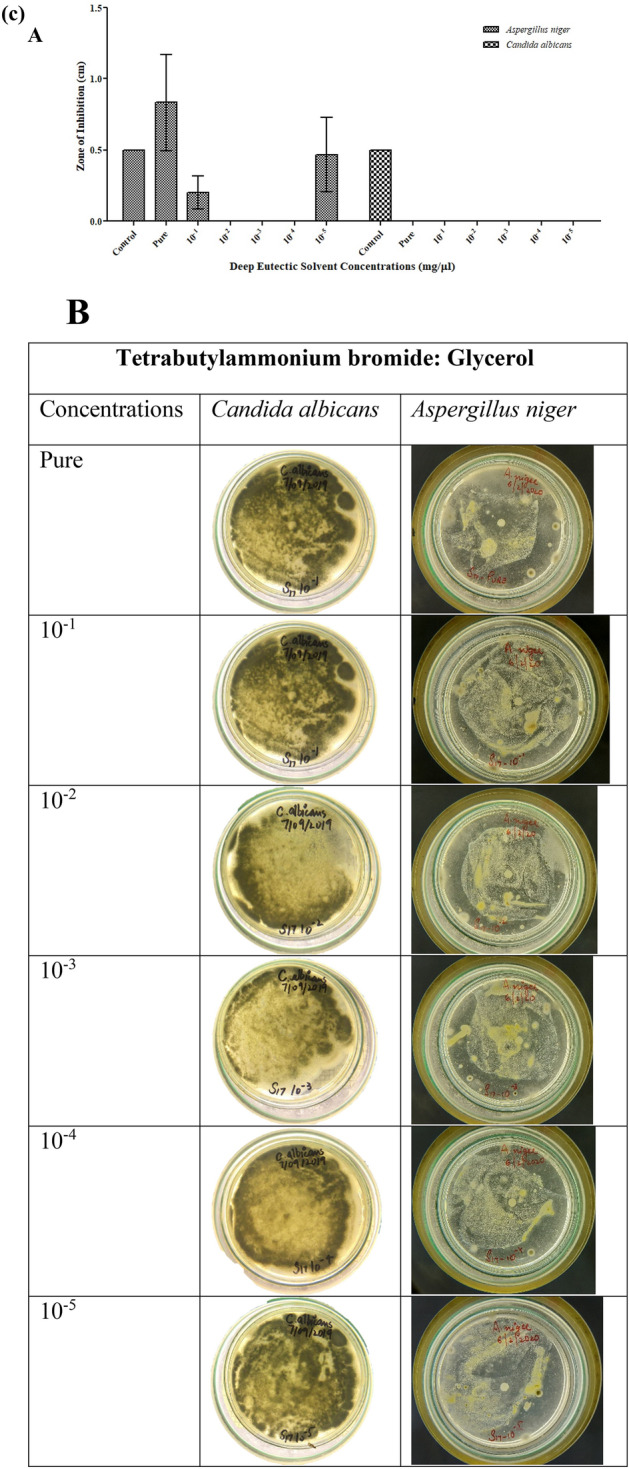
(**a**) Antifungal activities of TBABG against *C. albicans* and *A. niger*. **A**. TBABG in different concentrations (pure, 10^−1^ to 10^−5^). The *p*-value < 0.05 was considered significant and represented by *, while ^x^ implied no statistical analysis was applied. **B**. Zones of inhibition presented by TBABG against both tested fungal strains. (**b**) Antifungal activities of TBABEG against *C. albicans* and *A. niger*. A. TBABEG in different concentrations (pure, 10^−1^ to 10^−5^). The *p*-value < 0.05 was considered significant and represented by *, while ^x^ implied no statistical analysis was applied. **B**. Zones of inhibition presented by TBABEG against both tested fungal strains. (**c**) Antifungal activities of TBABDG against *C. albicans* and *A. niger*. A. TBABDG in different concentrations (pure, 10^−1^ to 10^−5^). The *p*-value < 0.05 was considered significant and represented by *, while ^x^ implied no statistical analysis was applied. **B**. Zones of inhibition presented by TBABDG against both tested fungal strains.

For antifungal activity, it can be observed that DESs showed low inhibition at low concentrations (10^−2^ to 10^−5^) for both fungal strains (*A. niger* & *C. albicans*). Nevertheless, both fungal strains were susceptible to DESs at higher concentrations (pure and 10^−1^ diluted solvent). As shown in Table S1, the greatest inhibition zone formed by pure TBABEG against *A. niger* was 0.96 cm ± 0.29. Overall, TBABEG was more toxic for microbes than the other DESs TBABG and TBABDG (Figs. 3 and 4). Similar results were obtained in a study where deep eutectic solvents containing glycerol had lower or no toxic results on fungus compared to the solvents containing ethylene glycol^25^. According to Wikene et al.^14^ of all the microorganisms investigated in their study, *C. albicans* was the least responsive towards both NADES, citric acid: sucrose (CS) and malic acid:fructose: glucose (MFG) (1:200), implying that fungi are substantially less sensitive to organic acid-containing NADES than bacteria^14^. Similarly in another research by Juneidi et al.^17^, the ChCl-based DESs containing glycerol had no toxic effect on *A. niger* whereas the one containing ethylene glycol as a consentient was somewhat toxic^17^. Generally, DES formation results from ionic or van der Waals interaction which can affect the cellular surface, respiration for growth and spore germination^27^ which can be the case for maximum antifungal activity against *A. niger* by TBABEG and TBABDG. When DES is diluted, its components usually dissociate from each other or the interactions between the components become weak. A similar finding can be attributed to TBABG whose toxicity increased towards *C. albicans* upon dilutions. It can suggest that the individual components of these DES were more toxic for *C. albicans* than the DES itself. The solvents TBABG, TBABEG and TBABDG were shown to have different antimicrobial properties. The TBABG was more active against gram negative bacteria than gram positive bacteria while it presented the same antifungal activity against both tested fungal strains. TBABEG and TBABDG were found most susceptible towards gram-positive bacterial strains. In pure form, both TBABEG and TBABDG were toxic against *A. niger,* while all three DESs were almost harmless to *C. albicans.* The fungal strains used in the present study, *A. niger* and *C. albicans,* represent the most common class of fungal strains used in the industrial production of enzymes, biodiesel, and biofermentation processes^28–31^. Therefore, the toxicity assessments of DESs on these fungal strains (*C. albicans*) may open new horizons for future applications on an industrial scale by using DESs as solvents in enzyme production and biofermentation processes.

### Fish toxicity

**Figure 5:**
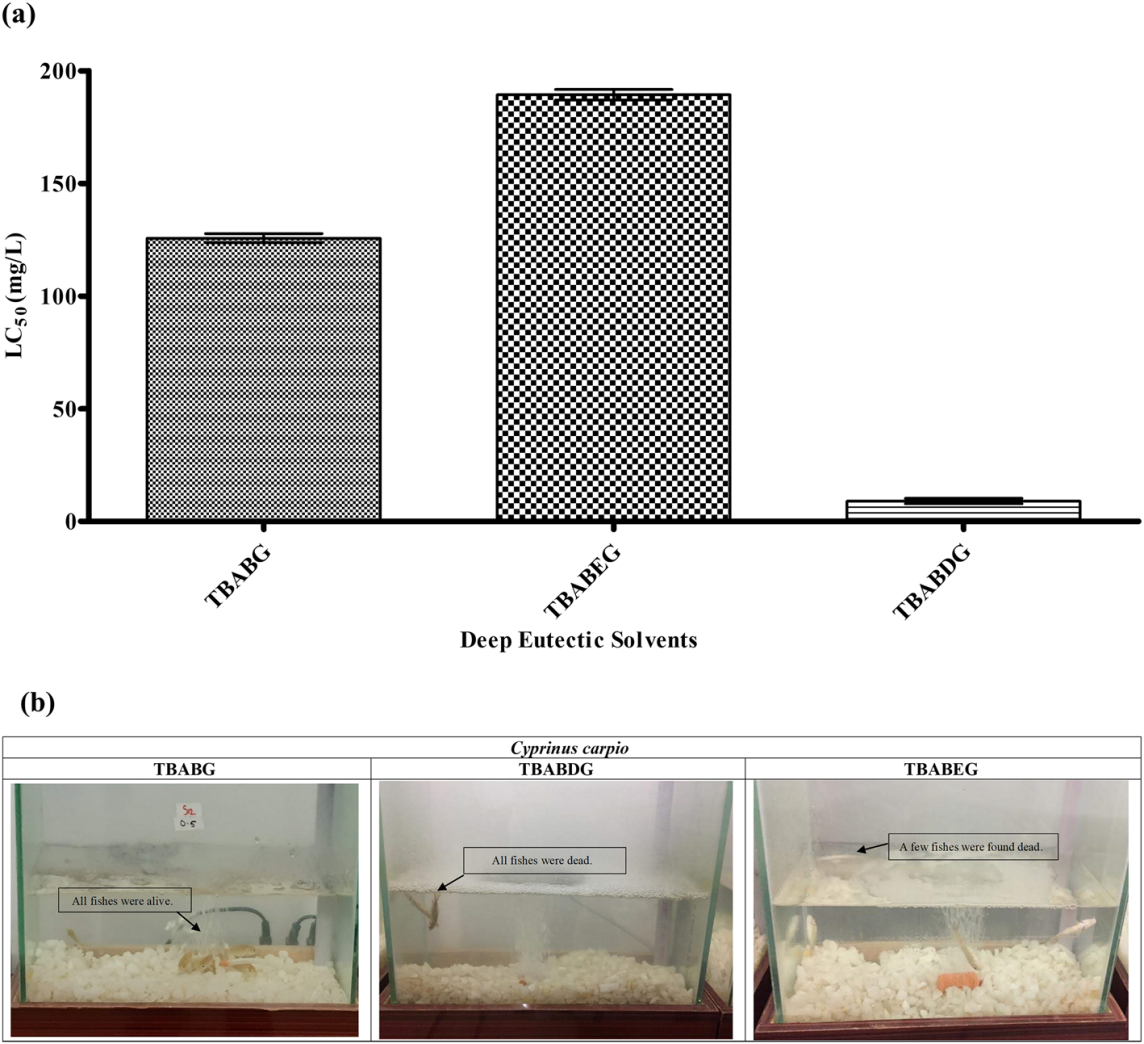
Fish toxicity analysis of synthesized DESs. (**a**) LC_50_ of deep eutectic solvents determined on *Cyprinus carpio* fish in water. (**b**) Dead and alive fishes are shown in tanks following exposure to respective DES.

According to the results obtained, two of the solvents i.e., TBABEG and TBABDG were subjected to a full test, and the LC50 value was calculated S3 that is presented with a 95% confidence interval (error bar) in Fig. 5a,b. The acute toxicity of DESs and their mixes for *C. Carpio* was determined following a 96-h exposure in a static setup. The tests were carried out following Organization for Economic Co-operation and Development (OECD) Guideline 203, with certain adjustments to account for the conditions^32^. According to the results listed in Tables S2 and , TBABG was found to be slightly toxic (10–100 mg/L) or relatively harmless as they showed no mortality during the limit test^33^. According to the OECD Guideline no. 203, DESs found to be particularly harmless were not subjected to a full test. Whereas, in the case of TBABEG and TBABDG where mortality occurred during the limit test, a full test was performed with five different concentrations. The lethal concentration was 190 mg/L and 7 mg/L for TBABEG and TBABDG, respectively. These results were supported by another study that investigated the LC50 values of ChCl-based DES with glucose and glycerol against the CCO fish cell line, and it was found to be > 2000 mg/L^34^. Other criteria, including temperature, pH, and water conductivity, were also measured and maintained during the test. During the experimental period, all the containers under observation noticed a slight increase in pH. The rise in pH was basically because of the discharged matter of the fish that is known to be alkaline. The pH values recorded during the experimental period were within the preferred range of 4 to 8. Almost all the containers maintained their normal conductivity between 7 and 7.5 mS cm^−1^, including the control ones (Table S3). According to fish toxicity analysis, TBABDG was the most toxic DES with 7 mg/L LC_50_, preceded by TBABG and TBABEG with LC_50_ of 126 mg/L and 190 mg/L, respectively. In the current study, common carp (*C. carpio*) was used because it was the most recommended fish type for toxicity analysis by OECD guidelines. The toxicity assessment of DESs on *C. carpio*, being the largest freshwater teleost, would be useful for their future involvement in the aquaculture industry whose main production region is present in Asia (~ 70%)^35^.

### Cytotoxicity assay

**Figure 6:**
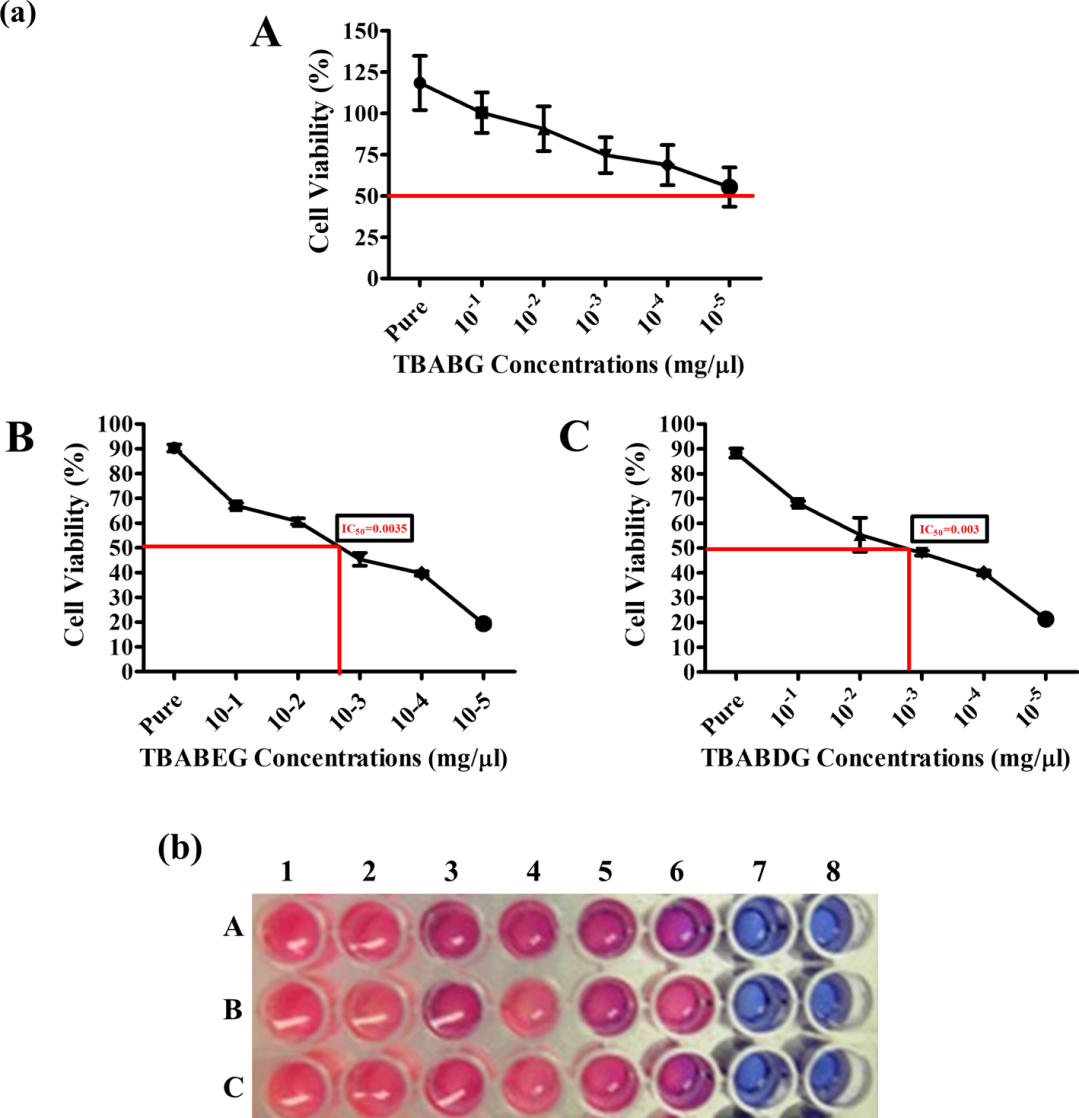
Cytotoxicity analysis of synthesized DESs. (**a**) Cell viability percentage of DESs for NTH313 cells after 72 h of exposure. Results are presented as mean ± SEM of triplicate experiments. **A.** TBABG, **B.** TBABEG, and **C.** TBABDG. **(b)** Variation in colours of AlamarBlue dye upon exposure to synthesized DESs. A, B and C represented TBABG, TBABEG and TBABDG, respectively. Lane 1 = Positive Control, Lane 2 = Pure DES, Lane 3 = 10^−1^ dilution, Lane 4 = 10^−2^ dilution, Lane5 = 10^−3^ dilution, Lane 6 = 10^−4^ dilution, Lane7 = 10^−5^ dilution, Lane 8 = Negative Control.

The in vitro toxicity was determined by culturing human fibroblast growth cell lines onto the membranes then a cell proliferation assay was done using Alamar blue. The experiment was conducted over three days, where DESs were co-cultured with cells, and then the results were compared with the control. The results were recorded by measuring the variation in absorbance due to different color changes. The color of alamarblue active ingredient changed from blue (resazurin) to bright red (resorufin) when it is reduced upon entering into cells, it is a quantitative indicator of cell viability which continuously reduce resazurin to resorufin. The results had been presented in Fig. 6b where the reduction rate had been decreased by increasing the dilution factor of the respective DES from bright red to blue (Fig. 6b). TBABG shows a greater increase in absorbance as compared to the control. TBABEG solvents show less cell proliferation than control (Table S4) (Fig. 6). Tetrabutylammonium bromide-based DES has not yet been assessed for its toxicity on any organism. However, tetrabutylammonium chloride-based DESs were assessed for their toxicity on Human cell lines in 2019 by Macario et al. They used two cell lines as a skin model (keratinocytes HaCaT and tumor melanocytes MNT-1) and tested its capabilities in the cosmetic and pharmaceutical sectors. There were significant results when three HBDs (hexanoic and butanoic acid, ethylene glycol, 1-propanol, and urea) and HBAs (cholinium chloride, tetramethylammonium chloride, and tetrabutylammonium chloride) were assessed for common viability. Tetrabutylammonium chloride-based DES, however, showed cytotoxicity for both cell lines, and cholinium chloride and tetramethylammonium chloride-based DES showed good biocompatibility^36^. All the DESs can be considered non-cytotoxic, according to ISO-10993-5-2009 guidelines, compounds or extracts which have < 70% viability can be categorized as toxic compounds. In a former study by Jover et al*.*, the alcohol based compounds were found less cytotoxic (IC_50_ 125–819 mM) although their toxicity was higher in human cell lines than in rodent cell lines^37^. Moreover, in the present study, a gradual increase in the cytotoxicity as there might be a decrease in the reduction of resazurin-dye due to low cellular metabolism by an increase in the water content of DESs. It can be hypothesized that due to charge delocalization by hydrogen bonding the deep eutectic solvents were less toxic in the culture medium. Having said that, some DESs remain intact and usually do not dissociate after passing through the cell membranes as they present low IC_50_ values than aqueous solutions of DESs. These results were in line with a study that also indicated that aqueous mixtures of two constituents of DESs were more toxic than pure DESs^17^. In the case of cell line proliferation, TBABG exhibits a greater cell viability percentage (118%) compared to the control while TBABEG (~ 85%) and TBABDG (94%) solvents demonstrated less cell growth. Although there are variations in cell viabilities, all three DESs can be attributed as non-toxic and it is recommended to assess their cytotoxicity against human cell lines. Moreover, these DESs can also be tested on animal models in future to ascertain their toxicities.

### Antioxidant activity assays

**Figure 7:**
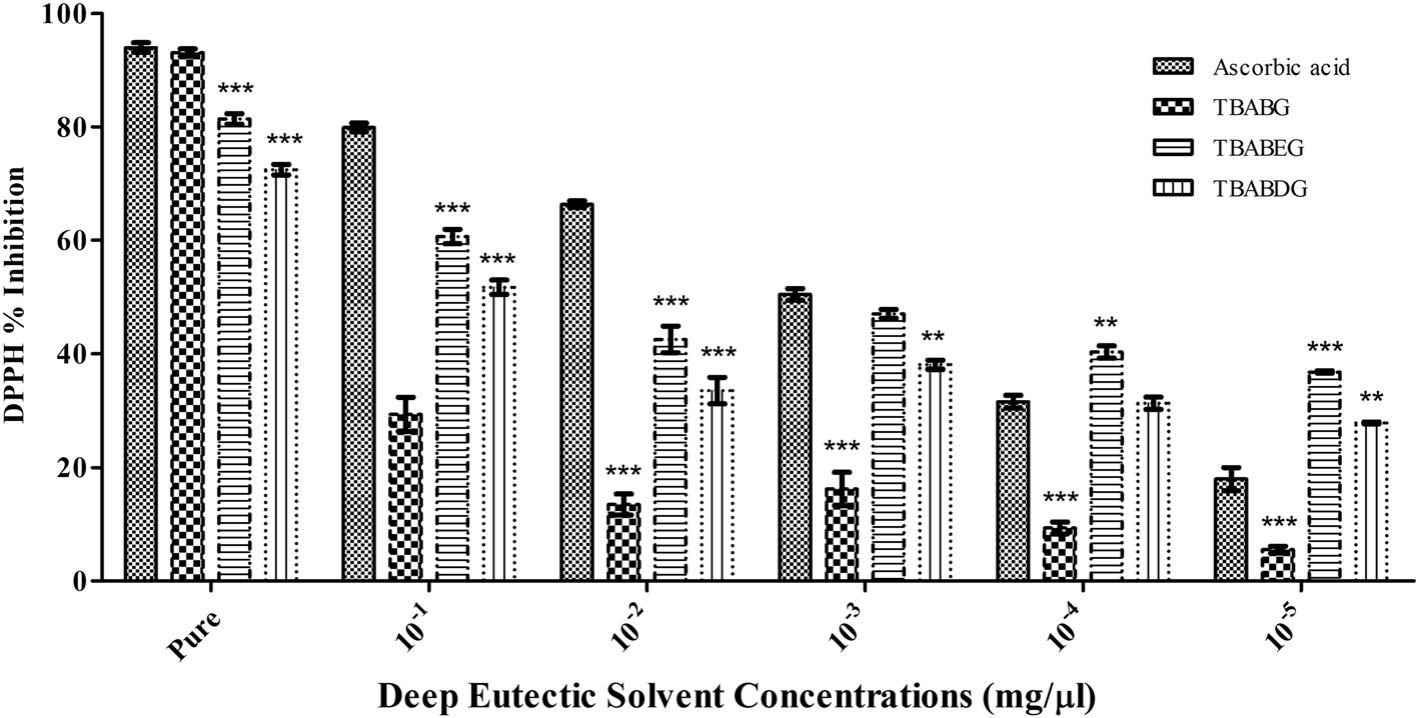
Antioxidant activity of synthesized DESs. Antioxidant % inhibition in different proportions from pure sample to 10^−1^–10^−5^ dilutions.

Although the antioxidant activity of NADES is less well-known and understood, various studies have been published on the subject. The power to scavenge the stable free radical 2, 2-diphenyl-1- picrylhydrazyl (DPPH) assay was performed based on the method described by previous research studies^38,39^. The theory behind DPPH Assay is that the hydrogen donor is an antioxidant. A stable free radical of DPPH is provided in a crystalline form where it donates its hydrogen atom to unstable molecules and inhibits oxidation^40^. The antioxidant activity of the test sample and ascorbic acid (as a standard) was measured spectrophotometrically, and the compound mixture showed a good DPPH absorption activity. The value obtained at 517 nm by the ultraviolet spectrophotometer at this wavelength absorbance by DPPH free radicals is strongest. The availability of a large number of free radicals leads to higher absorption. Lower absorbance will show less free radicals in the solution and higher inhibition capacity. Therefore, both antioxidant activity and absorbance are inversely related, whereas the disappearance of DPPH and antioxidant activity are directly related to each other. Antioxidant activities for the synthesized mixtures were evaluated via the DPPH method, which showed that pure TBABG had a higher inhibition percentage i.e., 93% followed by TBABEG having 80% and TBABDG having 74% of inhibition against free radicals as compared to standard Ascorbic acid which had 91.66% inhibition with 0.080 absorbances by UV spectrophotometer (Table S5). Absorption is increased when a significant number of free radicals are available. TBABG had more antioxidant properties against DPPH in comparison to TBABEG and TBABDG which may be due to their different polarities and more reactivity for DPPH anion (Fig. 7). This difference in DPPH scavenging activity implies that hydrogen bond donors would affect the properties of salt due to the formation of hydrogen bonding in their respective DESs. Antioxidants in the biological systems neutralizes the free radicals which had been formed by giving up their electrons in the oxidation reactions. As in the present study, TBABG was found to have more free radical inhibitory activity (93%) which was greater than the tested control. In the whole system, this can switch off the free radicals by donating its electrons, resulting in the breaking up of a chain reaction in the cells which can affect other cellular responses and their simultaneous affects on the whole system. Some antioxidants may act as damage control, not so protective, but they can induce cellular death by inducing apoptosis. Cell suicide sometimes needed to control the damage which can induce its effects at the body level^41^. A notable finding is that the antioxidant activity of the tetrabutylammonium bromide DES system with a gradual increase in water content slightly outperformed the pure DES system. Given this information, it can be concluded that the physiochemical properties of pure DESs may exert a big effect on the DPPH free radical scavenging effect. In line with the cytotoxicity analysis results, TBABG was found to have higher free radical percentage inhibition than other DESs. From these results, it can be hypothesized that the DES with higher antioxidant potential boosts up cellular metabolism and hence it presents a higher reduction in the resazurin dye which is suggestive of a high % of cell viability. These findings can be further implemented in future to analyse the effects of these DESs on the metabolomics of the cell along with the identification of related effects on metabolism such as oxidative stress, ammonia stress and DNA damage etc.

## Materials and methods

It has been confirmed that the experimental data collection complied with relevant institutional, national, and international guidelines and legislation with appropriate permissions from authorities of the College of Earth and Environmental Sciences Quaid e Azam Campus, University of the Punjab, Lahore, Pakistan. It has also been confirmed that the study was reported following ARRIVE guidelines.

### Chemicals

The chemicals purchased were as follows: Tetrabutylammonium bromide, Glycerol, Ethylene glycol, Diethylene glycol from Merck, Nutrient broth, Potato dextrose broth, yeast extract broth, MacConkey Agar, Mannitol Salt Agar, Potato dextrose agar, Ascorbic acid from Merck, Methanol from Sigma Aldrich, Sulphuric acid from Merck, Ammonium molybdate from Merck, Sodium phosphate from Merck, Dimethyl sulfoxide from Merck, and 2,2-Diphenyl-1-Picrylhydrazyl purchased from Merck having a commercial grade.

### Preparation of DESs

DESs were prepared by mixing dried salt (tetrabutylammonium bromide; TBAB) and hydrogen bond donor (glycerol; G, ethylene glycol; EG and diethylene glycol; DG) in stoichiometric proportions (1:2) (Table 1 and Fig. 1). The mixture was placed in an oil bath and heated at 60 °C for 20 min with continuous stirring until a homogenous mixture claimed to be Eutectic solvent was obtained.

**Table 1:** The selected deep eutectic solvents and their molar ratios.

Synthesized DESs	Abbreviation	Amount (g)	Molar Ratio
Tetrabutylammonium bromide: Glycerol	TBABG	1.61:0.92	2:1
Tetrabutylammonium bromide: Ethylene glycol	TBABEG	1.61:0.88	2:1
Tetrabutylammonium bromide: Diethylene glycol	TBABDG	1.61:1.06	2:1

### Fourier transforms infrared spectroscopy (FTIR) analysis

The synthesis of DES was confirmed by FTIR spectroscopy (FTIR, Nicolet 6700, USA) by using ATR mode in wave numbers ranging from 4000 to 400 cm^−1^ during 40 scans and the resolution of 8 cm^−1^.

### Toxicity assessment

The ethical committee of the College of Earth and Environmental Sciences, University of Punjab approved all the experiments. Experimental animals were handled in compliance with the Guide for the Care and Use of Laboratory Animals published by the US National Institutes of Health (NIH publication no.85-23, revised 1996). Cell lines were obtained as a gift from a repository of cell lines at IRCBM COMSATS University Lahore, Pakistan.

#### Antimicrobial activity assay

The test organisms required for this study were gram-negative (*E. coli;* ATCC#2592 and *P. aeruginosa;* NCTC#10662) and gram-positive (*S. aureus;* NCTC#6571 and *L. monocytogenes;* ATCC#19115) bacteria which were obtained from the Department of Microbiology and Molecular Genetics, University of the Punjab, Lahore. These strains were cultured in the Nutrient broth and were incubated at 37 °C for 24 h. The fungal strains chosen for this purpose were *A. niger *(ZGCL#1) and *C. albicans;* (ATCC#10231). These strains were cultured in the potato dextrose and yeast extract broth and were incubated at 37 °C for 96 h. The fungal strains of *A. niger* and *C. albicans* were obtained from the Research bank of Government College University, Lahore, Pakistan. The disk diffusion test was implied to assess the antimicrobial properties of deep eutectic solvents used in this study by measuring the diameters of the inhibition zones around DESs loaded disks. DES serial dilutions were prepared in 10^−1^–10^−5^ concentrations. 30 µl of the DESs solutions were loaded on the corresponding blank cellulose disks under sterile conditions. To analyze antibacterial activity, disks were incubated for 24 h on a MacConkey agar and mannitol salt agar plate seeded overnight inoculum of *E. coli* and *S. aureus,* respectively. For antifungal activity analysis, the disks loaded with DES solutions were incubated for 96 h at 37 °C on potato dextrose agar seeded with *A. niger* and *C. albicans*. Tetracycline discs were used to create a controlled group for bacterial strains, while Nystatin disc was used to create a control group for fungal strains. A pure group was formed by applying a pure solvent sample to the fungus^13^.

#### Fish toxicity assay

According to ISO 1982, the reconstituted water was prepared by which the pH, conductivity and hardness of water were 6.5–7.5, 7–7.5 mS cm^−1^ and 180–190 mg/L^42^. The *Cyrinus carpio* fish was chosen for their acute toxicity assay as a vertebrate model due to its important role in ecotoxicology. These fish were exposed to test DESs for 96 h. Firstly a limit test was performed at 100 mg/ml concentration then, if required, a comprehensive test was followed at 10–100 mg/ml concentrations. The toxicity tests were conducted to find the lethal concentration of DESs at which 50% of fish died. The six fish were added to the aquarium once each container had prepared the reconstituted water combinations. The fish were subjected to a 16-h daylight study during which they were not fed. Finally, control blank aquaria were used in the experiment. Each substance was tested three times, and mortalities were recorded at different time intervals, such as 1 h, 24 h, 48 h, 72 h and 96 h and then removed from the container. The results were referred to as the aqueous solutions of the DESs. Using US-Probit EP's analysis system, the lethal concentration of 50 was estimated based on the overall number of death counts. It was determined that LC 50 was less than 100 mg/L in cases when mortality occurred. However, if there was no mortality, the LC50 was calculated to be larger than 100 mg/L. Throughout the test, the quality and content of the water were monitored and documented^17^. MS Excel 365 ProPlus was used to examine the data. Probit analysis on MS Excel was used to calculate LC50s of the relevant solvents to assess the toxicity of DES on fish.

#### Cytotoxicity assay

Fibroblast cell lines (NIH/3T3) derived from mouse embryonic fibroblasts (ATCC#CRL-1658™) were cultured in Dulbecco’s modified Eagle’s medium (DMEM) supplemented with 100 g/ml of Penicillin/streptomycin (Sigma Aldrich, Life Sciences, USA) and 10% FBS (ThermoFisher Scientific, USA) and maintained in T75 culture flasks in the humidified atmosphere at 37 °C with 5% CO_2_. For the cytotoxicity assay, cells in the exponential growth phase (~ 90% confluency) with fresh medium changes every 2 to 3 days, were used. A hematocytometer and a microscope were used to count the cells on the day of seeding. Following dilution of 1:10 with DMEM media, NIH/3T3 cells were seeded in 24 well-plate at a density of 5 × 10^4^ per well in 100 μl of culture media. After overnight incubation, NIH/3T3 cells were treated with DES samples diluted in DMEM at different concentrations (0.1 µg, 1 µg, 10 µg, 100 µg and 1000 µg in 100 ml). Cells were grown on tissue culture plastic plates without liquid samples to compare the parameters of cells grown in the presence of liquid samples. After 72 h of exposure, 0.5 ml of 1 mM Alamar Blue solution was added to each well and further incubated for 3 h. A fluorescence plate reader (PR4100 Absorbance Microplate Reader BIO-RAD, UK) measured the absorbance at 570 nm. The percentage cell viability was calculated by using following formula,

$$\% Viability = \frac{{Absorbance_{sample} - Absorbance_{blank} }}{{Absorbance_{control} - Absorbance_{blank} }} \times 100$$

The IC50 values had been calculated by measuring absorbance at different DES concentrations.

#### Antioxidant activity

The free radical scavenging activity of deep eutectic solvents (TBABG, TBABEG and TBABDG) was estimated using stable free radical 2, 2-diphenyl-1- picrylhydrazyl (DPPH) assay. An aliquot of 1 ml of DES in methanol was mixed with 4 ml of 0.004% w/v of DPPH in methanol. Methanol and ascorbic acid were used as blank and standard^38^. Percent inhibition of free radical scavenging activity was calculated using the formula

$$DPPH\;Scavenging\;activity \left( \% \right) = \frac{{A_{Blank} - A_{DESSample} }}{{A_{Blank} }} \times 100$$

#### Statistical analysis

All the statistical analysis calculations and graphs were performed using Graph Pad Prism version 5.0 (GraphPad Software, San Diego, USA). The data values were represented as means ± standard error mean, and the *p*-value < 0.05 was considered statistically significant. To compare the results between groups, one-way ANOVA with Bonferroni post-hoc test was applied. All experiments were repeated three times.

### Ethical statement

It has been confirmed that the experimental data collection complied with relevant institutional, national, and international guidelines and legislation with appropriate permissions from authorities of the College of Earth and Environmental Sciences Quaid e Azam Campus, University of the Punjab, Lahore, Pakistan. It has also been confirmed that the study was reported following ARRIVE guidelines.

## Conclusion

The present study was done to synthesize three tetrabutylammonium bromide and alcohol-based deep eutectic solvents, namely TBABG, TBABEG and TBABDG. DESs prepared were excellent due to their homogeneous property and their formation was confirmed by FTIR analysis. According to the toxicity analysis, the antimicrobial potential of TBABG was lowest while TBABEG was found to be toxic against all tested bacterial and fungal strains. On the contrary, the LC_50_ of TBABEG was higher (190 mg/ml) than the other two DESs which makes it safer to use in aquaculture experiments. The cytotoxic and antioxidant analysis presented similar results as antimicrobial assessments. The TBABG was found less cytotoxic with maximum antioxidant potential than the other two DES. Conclusively, TBABEG can be found non-toxic within in vivo systems while TBABG would be safer within in vitro environment. The hydrogen bonding in the formation of DESs proved responsible for their toxicity level towards different organisms and cells.

## Supplementary Information

Supplementary Information.

## Data Availability

All data generated or analyzed during the study are included in the manuscript and its supporting file.

## References

[1] Stott PW, Williams AC, Barry BW (1998). Transdermal delivery from eutectic systems: Enhanced permeation of a model drug, ibuprofen. J. Control Release.

[2] Bi M, Hwang SJ, Morris KR (2003). Mechanism of eutectic formation upon compaction and its effects on tablet properties. Thermochim. Acta.

[3] Avula SG, Alexander K, Riga A (2010). Predicting eutectic behavior of drugs and excipients by unique calculations. J. Therm. Anal. Calorim..

[4] Dai Y, van Spronsen J, Witkamp G-J, Verpoorte R, Choi YH (2013). Natural deep eutectic solvents as new potential media for green technology. Anal. Chim. Acta.

[5] Zhang Q, Vigier KDO, Royer S, Jérôme F (2012). Deep eutectic solvents: Syntheses, properties and applications. Chem. Soc. Rev..

[6] Ge X, Gu C, Wang X, Tu J (2017). Deep eutectic solvents (DESs)-derived advanced functional materials for energy and environmental applications: Challenges, opportunities, and future vision. J. Mater. Chem. A.

[7] Smith EL, Abbott AP, Ryder KS (2014). Deep eutectic solvents (DESs) and their applications. Chem. Rev..

[8] Hayyan M (2016). Natural deep eutectic solvents: Cytotoxic profile. Springerplus.

[9] Abbott AP, Capper G, Gray S (2006). Design of improved deep eutectic solvents using hole theory. ChemPhysChem.

[10] Hayyan M (2013). Are deep eutectic solvents benign or toxic?. Chemosphere.

[11] Yusof R, Abdulmalek E, Sirat K, Rahman MBA (2014). Tetrabutylammonium bromide (TBABr)-based deep eutectic solvents (DESs) and their physical properties. Molecules.

[12] Zhao B-Y (2015). Biocompatible deep eutectic solvents based on choline chloride: Characterization and application to the extraction of rutin from *Sophora japonica*. ACS Sustain. Chem..

[13] Ghaedi H (2017). Toxicity of several potassium carbonate and phosphonium-based deep eutectic solvents towards *Escherichia coli* and *Listeria monocytogenes* bacteria. J. Environ. Anal. Toxicol..

[14] Wikene KO, Rukke HV, Bruzell E, Tønnesen HH (2017). Investigation of the antimicrobial effect of natural deep eutectic solvents (NADES) as solvents in antimicrobial photodynamic therapy. J. Photochem. Photobiol. B.

[15] Radošević K (2018). Antimicrobial, cytotoxic and antioxidative evaluation of natural deep eutectic solvents. Environ. Sci. Pollut. Res..

[16] Aryan R (2019). Expedient multicomponent synthesis of a small library of some novel highly substituted pyrido [2, 3-d] pyrimidine derivatives mediated and promoted by deep eutectic solvent and in vitro and quantum mechanical study of their antibacterial and antifungal activities. Mol. Divers..

[17] Juneidi I, Hayyan M, Mohd Ali O (2016). Toxicity profile of choline chloride-based deep eutectic solvents for fungi and *Cyprinus carpio* fish. Environ. Sci. Pollut. Res..

[18] Fahim AM, Farag AM (2020). Synthesis, antimicrobial evaluation, molecular docking and theoretical calculations of novel pyrazolo[1,5-a]pyrimidine derivatives. J. Mol. Struct..

[19] Yue D, Jia Y, Yao Y, Sun J, Jing Y (2012). Structure and electrochemical behavior of ionic liquid analogue based on choline chloride and urea. Electrochim. Acta.

[20] Mirghani MES, Kabbashi Nassereldeen A, Qudsieh IY, Elfaki FA (2008). A new method for the determination of toxic dye using ftir spectroscopy. IIUM Eng. J..

[21] Al-Majedy YK, Ibraheem HH, Jassim LS, Al-Amiery AA (2019). Antioxidant activity of coumarine compounds. Al-Nahrain J. Sci..

[22] Mirghani MES, Che Man YB (2003). Determination of hexane residues in vegetable oils with FTIR spectroscopy. J. Am. Oil Chem. Soc..

[23] Delgado-Mellado N (2018). Thermal stability of choline chloride deep eutectic solvents by TGA/FTIR-ATR analysis. J. Mol. Liq..

[24] Coates J, Meyers RA (2006). Interpretation of infrared spectra, a practical approach. Encyclopedia of Analytical Chemistry.

[25] Jangir AK, Lad B, Dani U, Shah N, Kuperkar K (2020). In vitro toxicity assessment and enhanced drug solubility profile of green deep eutectic solvent derivatives (DESDs) combined with theoretical validation. RSC Adv..

[26] Wen Q, Chen J-X, Tang Y-L, Wang J, Yang Z (2015). Assessing the toxicity and biodegradability of deep eutectic solvents. Chemosphere.

[27] Torri C (2011). Preliminary investigation on the production of fuels and bio-char from *Chlamydomonas reinhardtii* biomass residue after bio-hydrogen production. Biores. Technol..

[28] Salihu A, Bala M, Alam MdZ (2016). Lipase production by *Aspergillus niger* using sheanut cake: An optimization study. J. Taibah Univ. Sci..

[29] Malaquias JC, Regesch D, Dale PJ, Steichen M (2014). Tuning the gallium content of metal precursors for Cu(In, Ga)Se2 thin film solar cells by electrodeposition from a deep eutectic solvent. Phys. Chem. Chem. Phys..

[30] Kabbashi NA, Mohammed NI, Alam MZ, Mirghani MES (2015). Hydrolysis of *Jatropha curcas* oil for biodiesel synthesis using immobilized *Candida cylindracea* lipase. J. Mol. Catal. B Enzym..

[31] Jamal P, Saheed OK, Abdul Karim MI, Alam MZ, Muyibi SA (2015). A fermentative approach to ameliorating solid waste challenges within food and hospitality industry. Int. Biodeterior. Biodegrad..

[32] Oh J-H, Lee J-S (2014). Synthesis of gold microstructures with surface nanoroughness using a deep eutectic solvent for catalytic and diagnostic applications. J. Nanosci. Nanotechnol..

[33] Passino DRM, Smith SB (1987). Acute bioassays and hazard evaluation of representative contaminants detected in great lakes fish. Environ. Toxicol. Chem..

[34] Radošević K (2015). Evaluation of toxicity and biodegradability of choline chloride based deep eutectic solvents. Ecotoxicol. Environ. Saf..

[35] Hedayati A (2013). Acute toxicity test of mercuric chloride (Hgcl2), lead chloride (Pbcl2) and zinc sulphate (Znso4) in common carp (*Cyprinus carpio*). J. Clin. Toxicol..

[36] Macário IPE (2019). Cytotoxicity profiling of deep eutectic solvents to human skin cells. Sci. Rep..

[37] Jover R, Ponsoda X, Castell JV, Gómez-Lechón MJ (1992). Evaluation of the cytotoxicity of ten chemicals on human cultured hepatocytes: Predictability of human toxicity and comparison with rodent cell culture systems. Toxicol. In Vitro.

[38] Patil MP, Patil KT, Ngabire D, Seo YB, Kim GD (2016). Phytochemical, antioxidant and antibacterial activity of black tea (*Camellia sinensis*). Int. J. Pharmacogn. Phytochem. Res..

[39] Zhang H (2017). Gene expression and flavonol biosynthesis are induced by ultraviolet-B and salt stresses in *Reaumuria trigyna*. Biol. Plant..

[40] Alsaud N, Shahbaz K, Farid M (2021). Application of deep eutectic solvents in the extraction of polyphenolic antioxidants from New Zealand Manuka leaves (Leptospermum Scoparium): Optimization and antioxidant activity. J. Mol. Liq..

[41] Kaur S (2020). Antioxidant, antiproliferative and apoptosis-inducing efficacy of fractions from *Cassia fistula* L. leaves. Antioxidants (Basel).

[42] Barendsen GW (1982). Dose fractionation, dose rate and iso-effect relationships for normal tissue responses. Int. J. Radiat. Oncol. Biol. Phys..

