# Supplementary material for: In vivo and in vitro toxicity profile of tetrabutylammonium bromide and alcohol-based deep eutectic solvents
Source: Sci Rep. 2023 Jan 31;13:1777. doi: 10.1038/s41598-023-28928-y (PMC9889713; doi:10.1038/s41598-023-28928-y)
Supplement: Supplementary file 1 — Supplementary Information. [file 41598_2023_28928_MOESM1_ESM.docx]

Table S1: Antimicrobial activity (Zones of inhibitions, cm) of DESs with different dilution factors to *E. coli, P. aeruginosa, S. aureus, L. monocytogenes, A. niger and C. Albicans.*

| **DESs** | **Dilution factor** | ***E. coli*** | ***P. aeruginosa*** | ***S. aureus*** | ***L. monocytogenes*** | ***A. niger*** | ***C. albicans*** |
| --- | --- | --- | --- | --- | --- | --- | --- |
| **TBABG** | **Pure** | 0.10 ± 0.00 | 0.60 ± 0.26 | 0.05 ± 0.05 | – | 0.4 ± 0.06 | 0.46 ± 0.08 |
|  | **10^-1^** | 0.20 ± 0.00 | 0.30 ± 0.00 | 0.03 ± 0.03 | – | – | 0.53 ± 0.29 |
|  | **10^-2^** | 0.13 ± 0.01 | 0.20 ± 0.00 | 0.05 ± 0.05 | – | – | – |
|  | **10^-3^** | 0.25 ± 0.03 | 0.13 ± 0.03 | 0.13 ± 0.04 | – | – | 0.26 ± 0.26 |
|  | **10^-4^** | 0.10 ± 0.00 | 0.10 ± 0.00 | 0.13 ± 0.04 | – | – | – |
|  | **10^-5^** | 0.15 ± 0.03 | 0.07 ± 0.03 | 0.18 ± 0.07 | – | – | 0.53 ± 0.29 |
| **TBABEG** | **Pure** | 0.40 ± 0.00 | 0.40 ± 0.00 | 1.30 ± 0.00 | 1.35 ± 0.09 | 0.96 ± 0.29 | 0.23 ± 0.06 |
|  | **10^-1^** | 0.25 ± 0.00 | 0.20 ± 0.00 | 0.40 ± 0.06 | 0.60 ± 0.06 | – | 0.16 ± 0.03 |
|  | **10^-2^** | 0.15 ± 0.03 | 0.20 ± 0.00 | 0.45 ± 0.03 | 0.25 ± 0.14 | – | – |
|  | **10^-3^** | 0.15 ± 0.00 | 0.20 ± 0.00 | 0.35 ± 0.03 | 0.15 ± 0.09 | – | – |
|  | **10^-4^** | 0.20 ± 0.00 | 0.07 ± 0.03 | 0.20 ± 0.06 | 0.10 ± 0.06 | – | – |
|  | **10^-5^** | 0.20 ± 0.00 | – | 0.20 ± 0.12 | – | – | – |
| **TBABDG** | **Pure** | 0.17 ± 0.03 | 0.10 ± 0.00 | 0.33 ± 0.17 | 1.00 ± 0.00 | 0.83 ± 0.33 | – |
|  | **10^-1^** | 0.30 ± 0.00 | 0.17 ± 0.03 | 0.13 ± 0.09 | 0.13 ± 0.13 | 0.20 ± 0.11 | – |
|  | **10^-2^** | 0.17 ± 0.09 | 0.20 ± 0.00 | 0.13 ± 0.07 | 0.13 ± 0.09 | – | – |
|  | **10^-3^** | 0.20 ± 0.00 | 0.10 ± 0.00 | 0.13 ± 0.07 | – | – | – |
|  | **10^-4^** | 0.13 ± 0.03 | 0.30 ± 0.00 | 0.07 ± 0.03 | – | – | – |
|  | **10^-5^** | 0.15 ± 0.03 | 0.23 ± 0.09 | 0.03 ± 0.03 | 0.03 ± 0.03 | 0.46 ± 0.26 | 0.30 ± 0.30 |

Table S2: Toxicity estimation of synthesized deep eutectic solvents against *C. carpio* fish.

| **SYNTHESIZED SOLVENT** | **LC50 (mg/L)** | **Hazard Ranking*** |
| --- | --- | --- |
| Tetrabutylammonium bromide: G | 126±3.512 | + |
| Tetrabutylammonium bromide: EG | 190±4.041 | + |
| Tetrabutylammonium bromide: DG | 7.00±2.00 | ++ |

*Hazard ranking was done according to Passino and Smith^33^. ‘+’ indicates slightly toxic (10-100 mg/L) while ‘++’ indicates moderately toxic (1-10 mg/L) solvent concentration.

Table S3: Experimental parameters of fish during the limit test (100mg/L).

| **TBABG (S12)** | **Temp:** 31.5 | **Temp:** 31.5 | **Temp:** 31.3 | **Temp:** 31 | A full test was not required. |
| --- | --- | --- | --- | --- | --- |
|  | **pH:** 8.00 | **pH:** 8.16 | **pH:** 8.19 | **pH:** 8.21 |  |
|  | **Conductivity:** 371 | **Conductivity:** 368 | **Conductivity:** 378 | **Conductivity:** 402 |  |
| **TBABDG (S17)** | **Temp:** 31 |  |  |  | A full test was performed up to 0.1 mg/L. But all fishes were dead. |
|  | **pH:** 7.54 |  |  |  |  |
|  | **Conductivity:** 470 |  |  |  |  |
| **TBABEG (S18)** | **Temp:** 29 |  |  |  | A full test was performed up to 0.1 mg/L. But all fishes were dead. |
|  | **pH:** 7.88 |  |  |  |  |
|  | **Conductivity:** 423 |  |  |  |  |

Table S4: Percentage cell viability of human fibroblast growth factor cell lines against synthesized deep eutectic solvents.

| **DES** | **% Cell viability** | | | | | |
| --- | --- | --- | --- | --- | --- | --- |
|  | **Pure** | **10^-1^** | **10^-2^** | **10^-3^** | **10^-4^** | **10^-5^** |
| **TBABG** | 118.3±16.42 | 100.3±12.25 | 90.67±13.54 | 74.67±12.17 | 68.67±12.17 | 55.3±11.9 |
| **TBABEG** | 84.67±0.667 | 68.0±1.0 | 55.3±6.89 | 48.0±1.00 | 40.0±1.00 | 21.3±0.67 |
| **TBABDG** | 94.00±1.528 | 67.0±1.15 | 60.7±1.20 | 45.3±2.60 | 39.7±0.88 | 19.3±1.45 |
| **Control** | 100.0**±**0.5774 |  | | | | |

Table S5: Percentage inhibition of test samples by DPPH Assay.

| **DES** | **Percentage Inhibition** | | | | | |
| --- | --- | --- | --- | --- | --- | --- |
|  | **Pure** | **10^-1^** | **10^-2^** | **10^-3^** | **10^-4^** | **10^-5^** |
| **TBABG** | 93% | 29.4% | 13.5% | 16.3% | 9.3% | 5.5% |
| **TBABEG** | 80% | 60.7% | 42.53% | 47.02% | 40.3% | 36.8% |
| **TBABDG** | 74% | 51.7% | 33.6% | 38.1% | 31.3% | 27.9% |
| **Standard** | 91.66% | 79.9% | 66.35% | 50.4% | 31.61% | 17.9% |
